# Formulation and PEGylation optimization of the therapeutic PEGylated phenylalanine ammonia lyase for the treatment of phenylketonuria

**DOI:** 10.1371/journal.pone.0173269

**Published:** 2017-03-10

**Authors:** Sean M. Bell, Dan J. Wendt, Yanhong Zhang, Timothy W. Taylor, Shinong Long, Laurie Tsuruda, Bin Zhao, Phillip Laipis, Paul A. Fitzpatrick

**Affiliations:** 1 BioMarin Pharmaceutical, Novato, California, United States of America; 2 Department of Biochemistry and Molecular Biology, University of Florida, Gainesville, Florida, United States of America; Katholieke Universiteit Leuven Rega Institute for Medical Research, BELGIUM

## Abstract

Phenylketonuria (PKU) is a genetic metabolic disease in which the decrease or loss of phenylalanine hydroxylase (PAH) activity results in elevated, neurotoxic levels of phenylalanine (Phe). Due to many obstacles, PAH enzyme replacement therapy is not currently an option. Treatment of PKU with an alternative enzyme, phenylalanine ammonia lyase (PAL), was first proposed in the 1970s. However, issues regarding immunogenicity, enzyme production and mode of delivery needed to be overcome. Through the evaluation of PAL enzymes from multiple species, three potential PAL enzymes from yeast and cyanobacteria were chosen for evaluation of their therapeutic potential. The addition of polyethylene glycol (PEG, MW = 20,000), at a particular ratio to modify the protein surface, attenuated immunogenicity in an animal model of PKU. All three PEGylated PAL candidates showed efficacy in a mouse model of PKU (BTBR *Pah*^enu2^) upon subcutaneous injection. However, only PEGylated *Anabaena variabilis* (*Av*) PAL-treated mice demonstrated sustained low Phe levels with weekly injection and was the only PAL evaluated that maintained full enzymatic activity upon PEGylation. A PEGylated recombinant double mutant version of *Av*PAL (Cys503Ser/Cys565Ser), r*Av*PAL-PEG, was selected for drug development based on its positive pharmacodynamic profile and favorable expression titers. PEGylation was shown to be critical for r*Av*PAL-PEG efficacy as under PEGylated r*Av*PAL had a lower pharmacodynamic effect. r*Av*PAL and r*Av*PAL-PEG had poor stability at 4°C. L-Phe and trans-cinnamate were identified as activity stabilizing excipients. r*Av*PAL-PEG is currently in Phase 3 clinical trials to assess efficacy in PKU patients.

## Introduction

Phenylketonuria (PKU; OMIM 261600) is an autosomal recessive disease, which results in a toxic excess of Phenylalanine (Phe) due to the loss of function of phenylalanine hydroxylase (PAH; EC 1.14.16.1) with an incidence of 1:15,000 live births [1]. PAH catalyzes the irreversible conversion of the essential amino acid phenylalanine to tyrosine. In the absence of PAH, dietary phenylalanine is unable to be converted to tyrosine and undergo further processing to form key metabolites including L-dopa, thyroxine, dopamine, noradrenaline, adrenaline and melanin. In addition to high phenylalanine levels, which can lead to brain damage and impaired cognitive development, the additional decrease of key metabolites contribute to the PKU phenotype including attention deficit and albinism [2]. Since the 1960s, newborns have been tested for PAH enzyme deficiency and if positive, prescribed a low Phe diet [3]. Compliance to such a restricted diet is low and until recently remained the only treatment [4, 5]. However, not all PKU patients are completely deficient for PAH activity and a subset of patients respond to oral (6R)-L-erythro-5,6,7,8-tetrahydropiopterin (6R-BH4, sapropterin), the PAH cofactor [6, 7]. This subset comprises 30–50% of the PKU patient population, and many of those that respond to therapy continue to have elevated Phe levels, albeit lower than without sapropterin treatment [8].

Although attempts have been made to develop PAH as an enzyme replacement therapy [9], the need for the 6R-BH4 cofactor and the normal residence of this enzyme in the cytoplasm of hepatocytes have made PAH an improbable therapeutic [10]. In the 1970s, phenylalanine ammonia lyase (PAL; EC 4.3.1.24) from yeast or other non-mammalian sources was proposed as a therapy for the reduction of Phe for the treatment of cancer [11, 12]. In theory, depleting Phe would starve the cancer cells of this essential amino acid and inhibit proliferation. One issue regarding PAL treatment was the inherent immunogenicity of the foreign enzyme [13, 14]. Modification by conjugating PAL with polyethylene glycol (PEG) was attempted to reduce the immunogenicity. However, PEGylated adducts of PAL were shown to maintain immunogenic properties of PAL, even as the conjugate itself was a poor antigen to the immune response it generated [15]. PAL was first proposed as an enzyme substitution therapy for PKU through the use of extracorporeal enzyme reactors to remove Phe [16]. Although practical for proof of concept experiments, extracorporeal enzyme reactors or implanted PAL-coated shunts were deemed not practical for chronic care [17].

Obtaining sufficient quantities of plant and yeast PALs for use as a therapeutic were made possible by recombinant (r) expression in and purification from *E*. *coli* [18–20]. With adequate availability, rPAL as an enzyme substitution therapy could be evaluated.

Reducing serum phenylalanine through the depletion of Phe from the gut was attempted with orally administered rPAL: protected by enteric-coated gelatin capsules, silk fibroin, or by encapsulation in artificial cells or rPAL expressing bacteria [20–23]. However, these methodologies were minimally successful [24]. Subcutaneous administration of a PEGylated, more immunologically tolerable form of rPAL proved to be the most effective at reducing and controlling Phe levels in rodent PKU models [9, 25–27]. The conversion of serum Phe to trans-cinnamate and ammonia by rPAL is anticipated to reverse the hyperphenylalanine condition in PKU patients with the harmless metabolite, trans-cinnamate, being secreted in urine as hippurate [28].

Although PEGylated rPAL was shown to have reduced antigenicity, the PEGylation reaction generally reduced the activity of rPAL up to 75% [26]. A more effective therapeutic for a chronic indication would likely result by reducing antigenicity while maintaining full enzymatic activity [15, 25]. To this end, new PEGylation methodologies were developed, combined with testing PALs from different species as well as mutagenesis strategies to enhance PEG coverage and reduce the antigenicity of PAL [27, 29, 30]. Although most of the PAL enzymes explored as potential anti-cancer or PKU therapeutics were of yeast origin, PALs are also expressed in plants and bacteria. Early in the therapeutic research and development process, PAL from *Rhodosporidium toruloides* (*Rt*PAL) appeared the most promising. r*Rt*PAL is readily expressed at high titers in *E. coli [20]*. However, after exhaustive mutagenesis to modify the protein to reduce immunogenicity or to increase PEG coverage, r*Rt*PAL failed to maintain low Phe levels in repeat dose studies [30]. PALs from parsley, *Petroselinum crispum* (*Pc*PAL), yeast (*Rt*PAL) and two cyanobacteria, *Nostoc punctiforma* (*Np*PAL) and *Anabaena variabilis* (*Av*PAL) have previously been evaluated for short-term efficacy in the mouse model of PKU [30]. Sarkissian, et al., demonstrated the superiority of PEGylated wild type r*Av*PAL and specifically a double mutant of r*Av*PAL(C503S/C565S). Here, we detail the selection, PEGylation and formulation development, and *in vivo* characterization of PEGylated r*Av*PAL(C503S/C565S) (r*Av*PAL-PEG; wild type r*Av*PAL will be denoted as r*Av*PAL_WT_, e.g., r*Av*PAL_WT_-PEG throughout this paper). r*Av*PAL-PEG is currently undergoing clinical trials to demonstrate efficacy in reducing and maintaining low Phe levels in PKU patients [31]. r*Av*PAL-PEG represents a long awaited potential therapy for PKU patients.

## Materials and methods

### PAL purification and activity assay

Recombinant *Np*PAL, *Rt*PAL(R91K), *Av*PAL_WT_, *Av*PAL containing mutations to prevent aggregation, C503S/C565S, as well as 91 PALs from unidentified microbial origin were expressed from a pIBEX7 plasmid in the *E*. *coli* strains BLR or BL21(DE3) (EMD Millipore, Billerica, MA) (r*Np*PAL was co-expressed with the chaperonins GroEL and GroES) and purified by hydrophobic interacting chromatography followed by anion exchange chromatography [20, 30]. Briefly, the cell pellet was lysed in 25 mM Tris, 150 mM NaCl, pH 7.8 at OD_600_ = 100 by an APV homogenizer (SPX Corporation, Charlotte, NC) at 12,000 psi. Lysates were incubated at 60°C for 1–2 hours before cooling to 4°C and centrifugation to recover rPAL containing supernatants. The clarified supernatants were filtered through a Millistak+ charcoal filter (EMD Millipore, Billerica, MA). 25 mM Tris, 3 M (NH_4_)_2_SO_4_, pH7.8 was added to the filtered lysate at a ratio of 1:4 to obtain a final concentration of 0.6 M (NH_4_)_2_SO_4_ and then filtered through a 0.2 μm filter and loaded onto a hydrophobic interaction chromatography resin (ToyoPearl Butyl 650M; Tosoh Biosciences, San Diego, CA) and eluted with a gradient from 0.6 to 0 M (NH_4_)_2_SO_4_ in 25 mM Tris, pH 7.8. rPAL containing fractions were pooled and diluted with 20 volumes of 25 mM Tris, pH 7.8 and sterile filtered for anion exchange chromatography over a MacroPrep HighQ column (Bio-Rad, Hercules, CA). PAL was eluted with a linear NaCl gradient in 25 mM Tris, pH 7.8. The concentration of purified r*Av*PAL was determined by OD_280_ using an extinction coefficient of 0.75 (mg/mL)^-1^cm^-1^ (r*Rt*PAL = 0.51 (mg/mL)^-1^cm^-1^; r*Np*PAL = 0.83 (mg/mL)^-1^cm^-1^) and activity determined as previously described [32]. The PAL activity assay was initiated by the addition of 50 μL of rPAL into 950 μL of 100 mM Tris (pH 8.5) containing 22.5 mM Phe. The formation of the product, trans-cinnamic acid (t-CA), was monitored by optical absorption at 290 nm at 30°C and activity calculated based on the rate of t-CA formation; U = μM tCA/min [33]. The amount of t-CA synthesized was calculated using its extinction coefficient of 10,238 liter M^-1^cm^-1^.

### PEGylation

Purified rPALs were concentrated and buffer exchanged into 50 mM potassium phosphate, pH 8.5 via ultrafiltration/diafiltration (UF/DF) with 100K MWCO VivaFlow regenerated cellulose TFF cassette or VivaSpin concentrators (Sartorius, Goettingen, Germany). PEGylation reactions were performed with total N-hydroxysuccinimide-reactive (NHS) sites at a constant concentration of 2 mM with varying concentration of 20 kDa NHS-methoxyPEG (ME-200 HS; NOF America Corporation, White Plains, NY) to obtain molar ratios of 1:1, 1:1.6, 1:2, 1:2.4 and 1:3 available NHS-reactive sites per PEG molecule; e.g., r*Av*PAL has 17 lysines and an N-terminus available for modification by NHS-PEG to yield 18 potential reactive -NH_2_ sites. PEGylation reactions were performed in 50 mM potassium phosphate, pH 8.5 at room temperature for 3 hours. The reaction was quenched by adding an equal volume of formulation buffer, 25 mM Tris, 150 mM NaCl, pH 7.5 (TBS). Enhanced PEGylation was obtained by increasing the concentration of NHS-reactive sites to 4 mM and maintaining a 1:3 ratio. Unincorporated PEG was removed and PEGylated rPAL was formulated by extensive UF/DF with TBS using a 100K MWCO regenerated cellulose VivaFlow TFF cassette. This formulation was used in the animal studies described herein. PEGylation coverage was determined by subtractive peptide mapping by mass spectroscopy as previously described [29].

### *In vivo* evaluation of PEGylated PALs

Efficacy was assessed in BTBR *Pah*^enu2^ (PKU) mice by measuring plasma Phe concentration before and after scheduled injections of PEGylated rPAL at dosing and intervals indicated, as previously described [30, 34, 35]. The antibody response of the mice to r*Av*PAL was measured just prior to the first, third, fifth and seventh of 9 weekly injections and then 9 days following the final injection. Statistical significance of efficacy between rPALs was measured by a paired Wilcoxon signed rank test and for differentially PEGylated r*Av*PAL, by a repeated measures ANOVA with Bonferronni’s multiple comparison test using GraphPad Prism software (La Jolla, CA). Animal procedures were completed according to the guidelines for animal care at the University of Florida (Gainesville, FL) established by the Institutional Animal Care and Use Committee (IACUC approval # E452) with all efforts made to alleviate any distress. For studies requiring a large terminal blood sample (1 ml or more), mice were anesthetized with isoflurane. When unresponsive to pinch of belly skin with mouse-tooth forceps, the abdomen was opened, the inferior vena cava exposed, and a terminal exsanguination performed (2.5 ml syringe, 23 gauge needle), followed by cervical dislocation.

### Animal care

Mice were initially obtained (4 pairs) in 1997–1998 from Jackson Laboratories. A specific pathogen-free colony was maintained by in-house breeding (by the Laipis laboratory) in the University of Florida specific pathogen-free facility, using PKU male x HET female or HET male x HET female crosses. All mice were maintained with ad lib water and food. Breeder diet was used exclusively based on previous experience with PKU mice (the higher fat content improves PKU mouse condition). Animal Care Facility staff and veterinarians monitor animal health daily, but do not participate in breeding or experimental procedures; all mice were also monitored on a daily basis by Laipis lab personnel.

#### Analgesia and euthanasia

IACUC review of the protocols included veterinarian observation of bleeding from the tail vein; mice become accustomed to the very frequent procedure, 22 micoliter blood draw, and the reward of Phe-free chocolate, and no analgesia has been required. All mice in these studies were sacrificed as described (Isoflurane, terminal exsanguination, and cervical dislocation). Animals were removed from the study if physical condition deteriorated (not groomed, not social) or greater than 20% weight loss. These criteria were set based on discussion with ACF veterinarians, and careful comparison with untreated PKU mice.

#### Animal numbers and age, unexpected deaths

**Fig 1:**
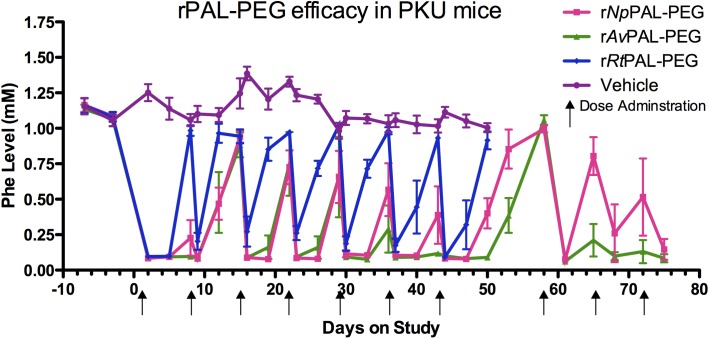
r*Av*PAL-PEG is superior in lowering and maintaining Phe levels in treated PKU mice. Male BTBR *Pah*^enu2^ mice (4 per group) were dosed weekly with 1U r*Np*PAL-PEG (3.3 mg), 2U r*Av*PAL-PEG (1 mg) or 6U r*Rt*PAL-PEG (3.75 mg) by sub-cutaneous injection. Blood was drawn just prior to drug administration, 1 day and 4 days following injection and analyzed for plasma Phe concentration. The non-responding r*Rt*PAL-PEG and vehicle control groups were discontinued at the end of a planned 7 week study. After a short discontinuation, the r*Np*PAL-PEG and r*Av*PAL-PEG mice resumed treatment for an additional three doses. The mean plasma Phe lowering effect by r*Av*PAL-PEG was significantly more efficacious than r*Rt*PAL-PEG (p< 0.0001) through 7 weeks and r*Np*PAL-PEG (p = 0.0224) for the complete study duration as determined by a Wilcoxon matched pairs test.

**Fig 2:**
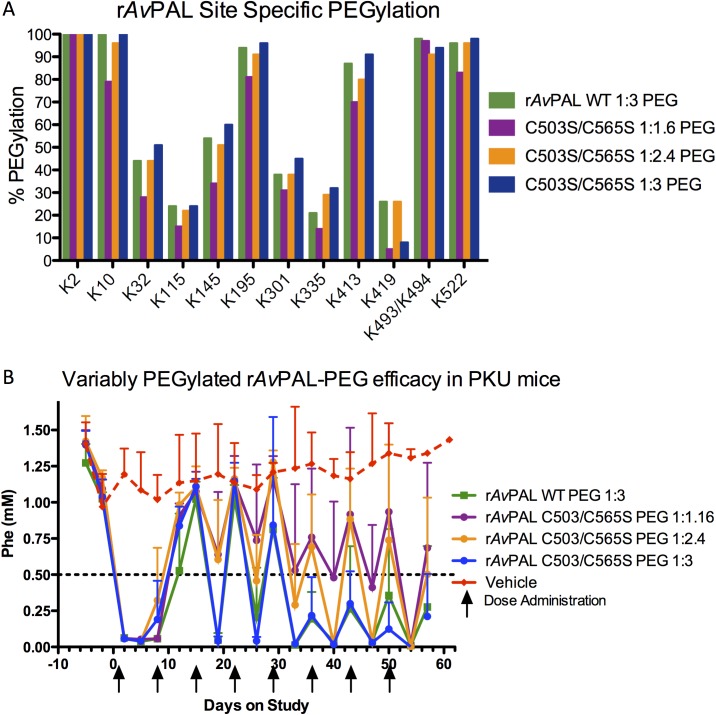
Efficacy of r*Av*PAL-PEG is dependent upon PEGylation ratio. (A) Three different PEGylation conditions resulted in minimal differences in PEGylation coverage as measured by subtractive peptide mapping. (B) Increasing the PAL:PEG ratio increases efficacy of r*Av*PAL-PEG in PKU mice (n = 4 male mice per condition).

Fig 1: 16 male mice, in three treatment groups plus 4 untreated controls; mice ranged from 14–40 weeks of age at the start of the experiment. Two mice were lost from the study; one mouse at 32 weeks of age (pancreatic hemangiosarcoma, 1 week before study end) and one at 25 (accidental death during handling, 5 weeks into study). Fig 2B: 18 male mice, in four treatment groups plus two untreated controls; mice ranged from 15–21 weeks at start of study. No mice were lost from this study.

### Antibody titer determination

Serum r*Av*PAL-specific IgG antibody levels were measured by an ELISA assay with r*Av*PAL as the capturing antigen. Anti-r*Av*PAL IgG was detected with biotinylated goat anti-mouse IgG antibody (Jackson ImmunoResearch Laboratories, West Grove, PA) and streptavidin-HRP conjugates (Thermo Scientific, Waltham, MA). All serum samples were initially screened at a 1:50 dilution. A cutpoint was generated for each assay plate based on normal mouse sera concurrently tested on the plate and any samples with an absorbance lower than the cutpoint generated by the normal mouse sera controls were reported as "<50" as the titer, i.e. negative. Any samples that had absorbance higher than this cutpoint during the initial screening were considered positive for IgG antibody and further diluted in a 1:3 series. The highest dilution factor for a specific sample that had absorbance above the cutpoint was reported as the titer for that sample. Since the reported value (titer) represents the last dilution at which antibody was still measurably above cutpoint, it is a unitless number. Day –2 samples (pre-dose) were only analyzed at a 1:50 dilution and so are reported here only as 50, <50 or >50.

### r*Av*PAL-PEG antigenicity

An Octet QK instrument (ForteBio, Menlo Park, CA) was used to measure antibody binding to r*Av*PAL PEGylated at various ratios. Affinity purified rabbit anti-r*Av*PAL polyclonal antibodies (BP-86) were biotinylated at pH 7.4 in PBS with NHS-LC-LC-Biotin (Thermo Scientific) at a 1:3 molar ratio. r*Av*PAL was PEGylated at 2:2, 2:4, 2:6 and 4:12 (mM -NH_2_: mM NHS-PEG) as described above. Anti-r*Av*PAL-Biotin was immobilized on a Streptavidin Octet biosensor at 5 μg/mL and then dipped into r*Av*PAL-PEG at 0.8 mg/mL and rate of association as determined by RU signal change was measured. Given the constant concentration of r*Av*PAL in the assay, it was hypothesized that increased PEG coverage would block epitopes and reduce Ab binding. As association kinetics correlate to concentration, the binding of differentially PEGylated r*Av*PAL-PEG could be measured and any decrease in association rate would correspond to a decrease in antigenicity.

### r*Av*PAL-PEG formulation development

Potential activity-stabilizing excipients were added as a concentrate following UF/DF and r*Av*PAL-PEG concentration. r*Av*PAL-PEG was buffer-exchanged into TBS and a 100X concentrate of excipient in TBS was added to the concentrated r*Av*PAL-PEG. Formulated r*Av*PAL-PEG (9–20 mg/mL) was aliquoted to 2 mL glass vials and underwent accelerated stability testing at various temperatures. Temperature was maintained by storing vials in dry incubators and laboratory refrigerators. At various time points, 10 μL was removed from each vial and transferred to 190 μL TBS to obtain a 1:20 dilution used in activity assays. Using the measured k_decay_ from at least two different accelerated storage temperatures, the activation energy of decay (E_a_) was derived from an Arrhenius plot (ln(-k_decay_) vs 1/T) with E_a_ = -slope • R (R = 8.314 J•T^-1^•mol^-1^), and the extrapolated k_decay_ at 4°C was calculated using the following equation:

$$k_{2}=\frac{k_{1}}{e^{\frac{E_{a}}{R}(\frac{1}{T_{2}}-\frac{1}{T_{1}})}}$$

Where k_1_ is k_decay_ obtained at T_1_ (e.g., 25°C or 298K) and k_2_ is the extrapolated k_decay_ at the proposed storage temperature of interest, T_2_ (e.g., 4°C or 277K) [36]. Data from multiple experiments sharing a common excipient were graphed together and the mean E_a_(decay) and standard error of the mean (SEM) calculated with Graphpad Prism software.

## Results

### r*Av*PAL(C503S/C565S) retains activity upon PEGylation

In Wang, et al., we described the structural and biochemical characteristics of r*Av*PAL_WT_ and a mutant form of r*Av*PAL with two mutated cysteines, Cys503 and Cys565 [32]. r*Av*PAL(C503S/C565S), hereafter referred to as r*Av*PAL, had similar specific activity and k_cat_/K_m_ to r*Av*PAL_WT_ (Table 1, Wang, et al., 2008). r*Av*PAL expression as soluble protein in *E*. *coli* was also greater than both r*Np*PAL and r*Rt*PAL (Table 1). r*Av*PAL (WT and mutant) titers ranged between 10–15 g/L whereas r*Rt*PAL expressed from the same plasmid ranged from 5–9 g/L and r*Np*PAL, even with the help of co-expressed chaperonins, GroEL/ES, never reached greater than 0.275 g/L. Whereas PEGylation of either r*Rt*PAL or r*Np*PAL resulted in ~66% activity loss, PEGylation of either WT or mutant r*Av*PAL resulted in no activity loss and no change in kinetic parameters.

**Table 1:** Characterization of recombinant PALs.

Candidate	Titers (g/L)	Specific Activity (Pre-PEGylation)	Specific Activity (Post-PEGylation)	K_m_ (mM)	k_cat_ (s^-1^) (per active site)	k_cat_/K_m_
r*Rt*PAL(R91K)	5–9	4.8 IU/mg	1.6 IU/mg	1.1	4	3.6
r*Np*PAL*	0.275	0.9 IU/mg	0.3 IU/mg	0.05	0.49	9.8
r*Av*PAL_WT_	10–15	2.0 IU/mg	1.6–2.0 IU/mg	0.06	1.15	19.2
r*Av*PAL(C503S/C565S)	10–15	2.0 IU/mg	1.6–2.0 IU/mg	0.05	1.0	20
r*Av*PAL(C503S/C565S)-PEG			1.6–2.0 IU/mg	0.05	1.2	24

*r*Np*PAL co-expressed with GroEL/ES to enhance expression

### r*Av*PAL-PEG has superior *in vivo* efficacy

In Sarkissian et al., we compared the effects of PEGylated recombinant *Rt*PAL(R91K), *Pc*PAL, *Np*PAL and *Av*PAL_WT_ to facilitate a reduction of Phe in PKU mice treated twice a week for 12 days [30]. In the studies presented here, Fig 1 shows the superiority of the double mutant r*Av*PAL-PEG over r*Rt*PAL(R91K)-PEG (p < 0.0001) and r*Np*PAL-PEG (p = 0.1858) by the sustained lowering of Phe in PKU mice dosed weekly for 6 weeks. When including data for r*Av*PAL-PEG and r*Np*PAL-PEG following a 2 week treatment holiday, with doses resuming at week 8, r*Av*PAL-PEG attains significant efficacy superiority over r*Np*PAL-PEG (p = 0.0224). Stabilization of Phe levels occurred in the fifth week of treatment for mice treated with r*Av*PAL-PEG, whereas mice treated with r*Rt*PAL(R91K)-PEG had Phe levels that returned to pre-dose levels within a week following treatment. r*Np*PAL-PEG treated animals had a longer and successive lowering of Phe levels than r*Rt*PAL(R91K)-PEG (p < 0.0001), but never reached the same level as the r*Av*PAL-PEG treated mice. Following dose discontinuation, r*Av*PAL-PEG mice quickly returned to sustained, low Phe levels upon resuming treatment.

In addition to the cyanobacterial PALs tested here and the plant and yeast PALs previously reported [30], we evaluated a number of other putative PALs from Diversa (San Diego, CA). Ninety-one PALs, derived from microbial organisms, were tested for recombinant expression in *E*. *coli*. Approximately 60 were soluble or partially soluble. Four were soluble, stable and demonstrated PAL activity. However, upon PEGylation, these rPALs were inferior to r*Av*PAL-PEG in lowering Phe in the PKU mouse.

### Optimizing PEGylation ratio

Gamez et al. had shown that the *in vivo* efficacy of PEGylated r*Rt*PAL was dependent upon PEGylation type and levels [25]. To understand the relationship between PEGylation levels and the effectiveness of r*Av*PAL-PEG on Phe lowering, r*Av*PAL was PEGylated at a ratio of 1:1.6, 1:1.24 and 1:3 (moles R-NH_2_: moles NHS-PEG) and tested in PKU mice. It had been shown that PEGylation levels were important for reducing immunogenicity while enhancing Phe-lowering ability [25, 26, 30]. It was clear that in the absence of PEGylation, rPAL is not effective at lowering Phe *in vivo*. The three r*Av*PAL PEGylation ratios tested had similar specific activities (1.3 ± 0.15 U/mg), yet small, but detectable differences in levels of lysine conjugation (Fig 2A). It should be noted that not all potential PEGylated peptides could be resolved, so differences might exist that were not detectable. These three test articles along with PEGylated (1:3) r*Av*PAL_WT_ were administered by subcutaneous injection to PKU mice weekly for 8 weeks and the plasma Phe levels were monitored as described in the Methods section. Although the overall PEGylation levels were similar as measured by subtractive peptide mapping, the increased level of PEG coverage, corresponding with increases of PEG in the reaction, resulted in significantly greater Phe-lowering activity *in vivo* (p < 0.002) (Fig 2B).

r*Av*PAL PEGylated at the ratios of 2:2, 2:4, 2:6 and 4:12 (mM NHS-reactive sites: mM PEG) were evaluated for their reactivity to anti-r*Av*PAL antibodies in a label-free binding assay using a Forte Bio Octet QK instrument (Table 2). Decreased antigenicity was observed with increased PEGylation. Part of the superior efficacy of the 1:3 PEGylated enzyme may be due to the decrease in immunogenicity (Table 3) and antigenicity (Table 2). The more heavily PEGylated enzyme was more effective *in vivo*, elicited a lower IgG response on average, and was less reactive to anti-r*Av*PAL antibodies *in vitro*.

**Table 2:** Decreased anti-r*Av*PAL-PEG IgG reactivity correlate to increased PEGylation ratio.

r*Av*PAL-PEG reactivity to anti-r*Av*PAL antibodies		
r*Av*PAL(C503S/C565S)	mM PAL: mM PEG	Relative k_assoc_ (RU/sec (1/10,000))
	Not PEGylated	26.08
	2:2	18.67
	2:4	4.25
	2:6	3.57
	4:12	1.58

**Table 3:** Decreased anti-r*Av*PAL IgG titers correlate to increased PEGylation ratio.

	r*Av*PAL antibody (IgG) titer, dilution factor					
PEGylated AvPAL Protein	Sample	Pre	D 15	D 28	D 43	D 64
WT (PEG 1:3)	S 1 01	<50	450	12150	4050	>1350
	S 1 06	<50	450	450	450	4050
	S 1 10	<50	50	50	150	450
	S 1 17	<50	150	450	450	1350
C565S/C503S (PEG 1:1.6)	S 2 02	50	450	12150	1350	1350
	S 2 07	<50	1350	12150	12150	36450
	S 2 11	<50	450	1350	12150	12150
	S 2 18	50	150	36450	26.57M	>36450
C565S/C503S (PEG 1:2.4)	S 3 03	<50	50	150	450	4050
	S 3 08	<50	50	50	50	450
	S 3 12	<50	50	150	450	4050
	S 3 13	<50	50	450	1350	4050
C565S/C503S (PEG 1:3)	S 4 04	<50	50	50	450	450
	S 4 09	50	50	50	150	450
	S 4 14	<50	<50	50	450	1350
	S 4 16	<50	<50	150	50	450
Vehicle	S 5 05	<50	<50	<50	<50	N/A*
	S 5 15	<50	<50	<50	<50	<50

*N/A: no serum sample for this time point

### Formulation development

**Fig 3:**
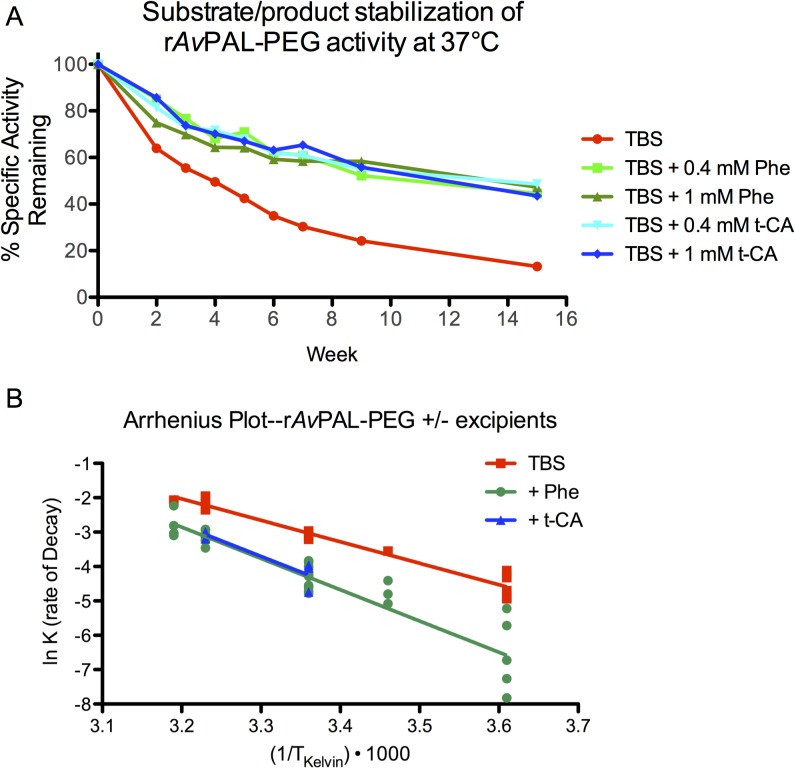
*Av*PAL substrate and product stabilize r*Av*PAL-PEG activity. L-Phe and trans-cinnamic acid (t-CA) were added to formulations of r*Av*PAL-PEG at varying concentrations, incubated at various temperatures and activity monitored over time. (A) A representative experiment is shown, where 12 mg/mL (0.2 mM) r*Av*PAL-PEG is formulated in the presence or absence of 0.4 and 1 mM Phe or t-CA, incubated at 37°C and activity monitored over time. A 2-fold molar excess of either t-CA or Phe is sufficient to improve activity retention. (B) An Arrhenius plot of the combined data from 7 experiments (S1–S3 Tables) with conditions at 4, 16, 25, 37, and 40°C.

An effective therapeutic must be stable. Whether a liquid formulation or lyophilized, it is preferred that a drug product have a reasonable shelf life. During development, it was observed that the activity of r*Av*PAL (PEGylated or not) would decrease over time in neutral-buffered saline solutions and could be accelerated by increasing temperature. To prevent enzymatic activity loss, buffer conditions and excipients were screened to identify activity-stabilizing agents. Both the *Av*PAL substrate, L-Phe, and enzymatic product, trans-cinnamic acid (t-CA), were identified as activity stabilizing excipients (Fig 3) in Tris-buffered saline (TBS).

Rates of decay were calculated for each condition from up to 7 independent experiments. The rates of decay (k_decay_) were plotted on an Arrhenius plot to obtain the energy of decay (E_a_ = -slope * R) and the extrapolated k_decay_ at 4°C calculated using the following equation:

$$k_{2}=\frac{k_{1}}{e^{\frac{E_{a}}{R}(\frac{1}{T_{2}}-\frac{1}{T_{1}})}}$$

Arrhenius plot analysis indicates that the T_90_ activity (time in which 10% of initial activity had been lost) of refrigerated formulations improved 6.5 fold in the presence of L-Phe or t-CA at 2–10 fold molar excess, improving the T_90_ from 13 weeks to 85 weeks for the experiment shown in Fig 3A. Combining data from 7 independent experiments (S1–S3 Tables), the T_90_ for r*Av*PAL-PEG increased from 11.3 weeks to 75.3 weeks for L-Phe containing formulations (Fig 3B and Table 4).

**Table 4:** Effect of excipients on r*Av*PAL-PEG activity rate of decay.

Formulation	-k_decay_ (40°C)	-k_decay_ (37°C)	-k_decay_ (25°C)	-k_decay_ (4°C)	E_a_(decay) (kJ/mol)	T_90_ 4°C (weeks)
**TBS**	0.1258, n = 1	0.1148±0.0082 n = 5	0.0457±0.0023 n = 4	0.0107±0.0018 (measured, n = 5) 0.0093 (calculated)	51.89 ± 2.84	11.3
**TBS + Phe (0.4–2.5mM)**	0.0592±0.0098n = 6	0.0438±0.0023 n = 9	0.0146±0.0013 n = 12	0.0019±0.0008 (measured, n = 6) 0.0014 (calculated)	75.64 ± 5.03	75.3
**TBS + t-CA (0.4-1mM)**	ND	0.0466±0.0029 n = 3	0.0151±0.0032 n = 3	0.0015 (calculated)	75.03 ± 16.47	70.2

r*Av*PAL-PEG at 9-20mg/mL was formulated in the presence or absence of the indicated excipients. Rates of decay (k_decay_ ± SEM) were calculated for each condition tested from up to 7 independent experiments (n represents number of independent samples). Combined data were used to calculate the energy of decay, E_a_(decay), and predict the T_90_ at 4°C using k_decay_ at 25°C. Average E_a_(decay) ± SEM was determined by the best-fit slope of all data plotted on an Arrhenius plot (Fig 3) as determined by a non-linear regression algorithm using Graphpad Prism software. All data used to generate average k_decay_ and E_a_(decay) values are shown in S1–S3 Tables. SEM = Standard Error of Mean.

## Discussion

The PKU patient has limited options for lowering their plasma Phe levels. A Phe restricted diet has been the standard of care for 50 years, but is unpalatable and compliance post adolescence is low [4, 5]. Some patients with residual PAH activity can enhance that activity by taking the orally available PAH co-factor, 6R-BH4, although many PAH-deficient patients are non-responsive to this therapy, and some who are responsive have insufficient Phe lowering [6, 8]. Those that do respond strongly have the luxury of expanding their diet beyond PKU-prescribed foods. Enzyme substitution therapy with PAL would serve as a treatment for all PAH-deficient patients and should allow for the liberalization of their diet. In the absence of treatment and compliance to a low Phe diet, systemic Phe concentrations become neurotoxic with symptoms ranging from mild cognitive impairment and behavioral disorders to severe mental retardation and microcephaly.

The development of PAL as a potential therapeutic enzyme substitution therapy for PKU has been postulated since the late 1970s, but has proven to be a difficult task [37]. To develop PAL as a therapeutic enzyme, four challenges were met: 1) identify a PEGylated PAL with good pharmacological characteristics; 2) optimize PEGylation to reduce antigenicity while maintaining therapeutic activity *in vivo*; 3) maximize production; and 4) ensure a long shelf life. Here we have described our selection of r*Av*PAL-PEG as the therapeutic candidate for treating PKU due to phenylalanine hydroxylase deficiency.

The inherent immunogenicity of the non-mammalian PAL enzyme has been a challenge to overcome in order to develop PAL as an enzyme substitution therapy for PKU [14]. Early experiments with PEGylated PAL from yeast showed that PEGylation could blunt, but not eliminate the immune response [15]. Choice of PEG, in terms of structure, size and vendor, and optimization of PEGylation methods were key factors to develop an effective therapeutic enzyme [25–27, 30]. We discovered that a 20 kDa linear mPEG from NOF was superior to smaller linear PEGs, branched PEGs and 20 kDa linear mPEG from a different vendor in protecting rPAL from an immune response [25, 27, 30]. Developing the proper PEGylation strategy reduced rPAL antibody titers in the mouse study presented, increased efficacy and ultimately reduced the cost of goods as the ratio of NHS-mPEG to -NH_2_ on rPAL became smaller due to an overall increased concentration of protein in the reaction [25, 30]. Although the antibody titers were reduced, antibodies to r*Av*PAL were generated and the molecule remains immunogenic. However, we observed reduced antigenicity of r*Av*PAL-PEG *in vitro* that correlates with levels of PEGylation. Thus, PEGylation serves to protect r*Av*PAL-PEG from the antibodies developed against r*Av*PAL, improving the pharmacokinetic and pharmacodynamic properties of r*Av*PAL-PEG. This observation is consistent with earlier studies [15].

Each of the rPAL-PEGs tested here showed a dramatic lowering of Phe following the first injection. However, Phe rose to baseline levels by the end of the first week for r*Rt*PAL-PEG and seven days after the second injection for r*Av*PAL-PEG and r*Np*PAL-PEG. Following repeat injection, Phe levels increased prior to the next injection, but for r*Av*PAL-PEG and r*Np*PAL-PEG the recovery of Phe levels was reduced with each subsequent injection leading to a sustained lowering after the fifth or sixth injection. The mechanism of this transient loss of pharmacodynamic effect in mice, although not fully understood, was hypothesized to be caused by a transient anti-PEG response that diminished rPAL-PEG activity and that as this response (most likely IgM-mediated) declined, allowed a persistence of active rPAL-PEG in serum. PEGylated protein and liposomes have been shown to elicit a transient IgM response and contribute to the accelerated blood clearance of PEGylated proteins [38, 39]. Although we could not directly measure the IgM responses, the transient nature of the reduced pharmacodynamic effect support the speculation of IgM involvement as such responses are commonly transient. Fig 1 shows that, at least with r*Av*PAL-PEG, simple drug accumulation could not be responsible for the improvement of the pharmacodynamic effect over time as drug treatment interruption followed by drug reintroduction did not restore the loss of pharamcodynamic effect. It is clear that not every PEGylated PAL was equally efficacious in this way as r*Rt*PAL-PEG was not able to overcome this clearance or neutralizing activity at all to maintain a low Phe level between injections, and r*Np*PAL-PEG was not as potent as r*Av*PAL-PEG in overcoming such response.

r*Av*PAL was superior to other rPALs tested in its ability to be expressed at high titer, maintain activity following PEGylation, and, upon PEGylation, showed significant efficacy in reducing and maintaining low Phe levels in the PKU mouse. Nonetheless, the process by which r*Av*PAL was selected as a therapeutic (post-PEGylation) was primarily an empirical search for the most efficacious PAL in reducing Phe *in vivo* after PEGylation, and it is challenging to identify specific properties that yielded such a superior response in the PKU mouse model. The N-terminal 54 amino acid extension and a 122 amino acid insertion conserved among plant and yeast PALs, but absent in bacterial PALs, was thought to be highly immunogenic [27, 29, 32, 40]. Extensive mutagenesis of these domains in r*Rt*PAL to reduce immunogenicity or by increasing PEG coverage was ineffective at reducing r*Rt*PAL-PEG immunogenicity or improving efficacy [30]. *Np*PAL and *Av*PAL lack these domains, and one can speculate that this contributes to the superiority of the cyanobacterial PALs to *Rt*PAL in reducing Phe levels in PKU mice when PEGylated.

A robust PEGylation strategy to minimize or deflect an immune response, while maintaining full enzyme activity was developed. Although only small differences in PEGylation levels were observed in the detectable reactive sites when r*Av*PAL was PEGylated at a ratio of 1:1.6, 1:2.4, or 1:3, there was an observable difference in efficacy and immune response, highlighting the correlation of PEG coverage with efficacy. It should be noted that many efforts were made to optimize PEG coverage on r*Rt*PAL, but r*Rt*PAL-PEG never obtained the same efficacy as r*Av*PAL-PEG even with enhanced PEG coverage through lysine additions [25, 27, 29, 30]. As was similarly observed by Ikeda with r*Pc*PAL, extensive PEGylation negatively affected enzyme activity in both r*Rt*PAL-PEG and r*Np*PAL-PEG with a reproducible 66% activity loss [26]. Surprisingly, r*Av*PAL activity was not reduced upon PEGylation. It was observed that r*Av*PAL, and not r*Rt*PAL nor r*Np*PAL, had associated t-CA upon purification as observed in the crystal structures and by reverse phase HPLC [32, 40, 41]. As the occupancy of the active site by t-CA stabilized r*Av*PAL activity and discussed below, the absence of bound t-CA in r*Rt*PAL and r*Np*PAL might have made these enzymes more susceptible to activity loss during the PEGylation reaction.

PAL catalyzes the β-elimination of ammonia from Phe through an electrophilic attack of the aromatic ring by the MIO prosthetic group (3,5-dihydro-5-methyldiene-4H-imidazol-4-one) in the PAL active site in a Friedel-Crafts-type reaction [41–43]. The MIO in PAL is highly susceptible to peptide fragmentation by reactive oxygen species (Erno Pungor, personal communication). To enhance long-term stability, protection of the MIO by binding PAL substrate or product was explored. It had been shown that Phe and t-CA, but not tyrosine could enhance the thermo-stability of r*Av*PAL [32]. The loss of activity was identified as an instability of the active site MIO prosthetic group in which hydrolysis led to an irreversible clipping of the protein and was protected by both Phe and t-CA (Dan Wendt and Erno Pungor, personal communication). As formulation excipients, both Phe and t-CA significantly stabilized r*Av*PAL-PEG activity, extending its T_90_ activity stability from 11 to 75 weeks at 4°C. As PAL catalyzed the conversion of Phe to t-CA, it is believed that t-CA remains in the active site until displaced by a new Phe molecule [32, 42]. In the absence of additional Phe, two of the four active sites of r*Av*PAL are occupied with t-CA [32]. In the presence of Phe, the ratio of t-CA binding increases and thus, the presence of either Phe, which is readily converted to t-CA, or t-CA act as activity stabilizing excipients protecting the MIO from reactive oxygen species and preserving the activity of r*Av*PAL-PEG.

PALs had traditionally been considered specific to plants and yeast. However, Moffit et al., identified two PALs from cyanobacteria that had lower molecular weights than PALs from plants or yeast and while more similar in size to bacterial histidine ammonia lyase (HAL; EC 4.3.1.3) they were structurally more similar to eukaryotic PALs [40]. One of these, *Av*PAL, was initially designated as a HAL (GenPept access #: YP_324488) based on sequence similarity and presumably on the lack of bacterial PAL precedent, but was reported to have no histidine ammonia lyase activity [40]. In total, rPALs from 95 species were tested for expression and PAL activity. In addition to evaluating wild type enzymes, some expressed and active rPALs were modified by mutagenesis to reduce immunogenicity, increase activity, modify antigenic epitopes, or enhance PEG coverage. Upon PEGylation, these rPAL-PEGs were tested in the PKU mouse model. The result of these studies (Fig 1 and [25, 27, 30, 32]) allowed for the selection of r*Av*PAL as the therapeutic candidate PAL for PKU.

The bacterial PALs tested appeared to have advantages over yeast and plant PALs. Possibly by lacking potential immune-reactive and protease-sensitive domains, both r*Np*PAL-PEG and r*Av*PAL-PEG had significantly better efficacy in lowering plasma Phe levels in the PKU mouse [30, 32, 40]. r*Av*PAL had a significant advantageous characteristic compared to r*Np*PAL; it could be expressed at ~40 fold higher levels in *E*. *coli*, allowing for a much less costly production. In addition, unlike many enzymes and even other PAL’s (including r*Np*PAL), r*Av*PAL recovered full enzyme activity even after extensive PEGylation. This is important because light PEGylation of r*Av*PAL (or any PAL) leads to reduced efficacy in the PKU mouse model. r*Av*PAL was susceptible to formation of soluble aggregates, but mutation of two surface cysteine residues abrogated the aggregation without affecting enzyme activity [32]. Ultimately, the best therapeutic candidate was produced by choosing the best species of PAL and the optimal extensive PEGylation method. Finally, the addition of Phe or t-CA to the formulation allows for a stable drug product with a long shelf-life. r*Av*PAL-PEG (pegvaliase) is currently undergoing evaluation in a Phase 3 clinical study to evaluate the safety and efficacy for the treatment of PKU.

## Supporting information

S1 Table r*Av*PAL-PEG Data for Arrhenius Plot (TBS). r*Av*PAL-PEG at 12 mg/mL was formulated in Tris-buffered saline (TBS). Rates of decay (k_decay_ ± SEM) were calculated for each condition tested from 5 independent experiments (n represents number of independent samples at each incubation temperature). The k_decay_ data are plotted on the Arrhenius Plot shown in Fig 3B. The energy of decay, E_a_(decay), was calculated for each study and the combined data (Average ± SEM) are shown in Table 4.
(DOCX)

S2 Table r*Av*PAL-PEG Data for Arrhenius Plot (TBS + Phe). r*Av*PAL-PEG at 9–20 mg/mL was formulated in Tris-buffered saline (TBS) with the indicated concentration of L-Phe. Rates of decay (k_decay_ ± SEM) were calculated for each condition tested from 7 independent experiments (n represents number of independent samples at each incubation temperature). The k_decay_ data are plotted on the Arrhenius Plot shown in Fig 3B. The energy of decay, E_a_(decay), was calculated for each condition and the combined data (Average ± SEM) are shown in Table 4.
(DOCX)

S3 Table r*Av*PAL-PEG Data for Arrhenius Plot (TBS + t-CA). r*Av*PAL-PEG at 12 mg/mL was formulated in Tris-buffered saline (TBS) with the indicated concentration of trans cinnamic acid (t-CA). Rates of decay (k_decay_ ± SEM) were calculated for each condition tested from 2 independent experiments (n represents number of independent samples at each incubation temperature). The k_decay_ data are plotted on the Arrhenius Plot shown in Fig 3B. The energy of decay, E_a_(decay), was calculated for each condition and the combined data (Average ± SEM) are shown in Table 4.
(DOCX)

## Abbreviations

PKU: phenylketonuria
PAH: phenylalanine hydroxylase
PAL: phenylalanine ammonia lyase
PEG: polyethylene glycol
Av: Anabaena variabilis
6R-BH4: (6R)-L-erythro-5,6,7,8-tetrahydropiopterin
r: recombinant
Rt: Rhodosporidium toruloides
Pc: Petroselinum crispum
Np: Nostoc punctiforma
r*Av*PAL-PEG: PEGylated r*Av*PAL(Cys503Ser/Cys565Ser)
UF/DF: ultrafiltration/diafiltration
TFF: tangential-flow filtration
TBS: Tris-buffered saline
t-CA: trans-cinnamic acid
MIO: 3,5-dihydro-5-methyldiene-4H-imidazol-4-one
