# Supplementary material for: Formulation and PEGylation optimization of the therapeutic PEGylated phenylalanine ammonia lyase for the treatment of phenylketonuria
Source: PLoS One. 2017 Mar 10;12(3):e0173269. doi: 10.1371/journal.pone.0173269 (PMC5345807; doi:10.1371/journal.pone.0173269)
Supplement: S2 Table — rAvPAL-PEG at 9–20 mg/mL was formulated in Tris-buffered saline (TBS) with the indicated concentration of L-Phe. Rates of decay (kdecay ± SEM) were calculated for each condition tested from 7 independent experiments (n represents number of independent samples at each incubation temperature). The kdecay data are plotted on the Arrhenius Plot shown in Fig 3B. The energy of decay, Ea(decay), was calculated for each condition and the combined data (Average ± SEM) are shown in Table 4. (DOCX) [file pone.0173269.s002.docx]

S2 Table. r*Av*PAL-PEG Data for Arrhenius Plot (TBS + Phe).

| **Study** | **Phe (mM)** | **Phe:PAL** | **-k_decay_ 40°C, n=6** | **-k_decay_ 37°C, n=9** | **-k_decay_ 25°C, n=12** | **-k_decay_ 16°C, n=3** | **-k_decay_ 4°C, n=12** | **E_a_(decay) kJ/mol** |
| --- | --- | --- | --- | --- | --- | --- | --- | --- |
| 1 | 1 | 5:1 |  | 0.0424 | 0.0133 |  | NMD ^1^ | 74.2 |
| 2a | 1 | 5:1 |  | 0.0389 |  |  | 0.0007 | 86.9 |
| 2a | 2.5 | 12.5:1 |  | 0.0315 |  |  | NMD |  |
| 2b | 0.4 | 2:1 |  |  | 0.0106 |  | NMD |  |
| 2b | 1 | 5:1 |  |  | 0.0145 |  | 0.0012 | 81.4 |
| 2b | 2 | 10:1 |  |  | 0.0084 |  | 0.0004 | 99.5 |
| 3 | 0.4 | 2:1 |  | 0.0467 | 0.0090 |  | NMD | 105.4 |
| 3 | 1 | 5:1 |  | 0.0398 | 0.0100 |  | NMD | 88.4 |
| 4 | 0.5 | 1.5:1 | 0.0478 | 0.0523 | 0.0182 | 0.0062 | 0.0054 | 50.4 |
| 4 | 1 | 3:1 | 0.0483 | 0.0436 | 0.0183 | 0.0121 | NMD | 45.2 |
| 4 | 2 | 6:1 | 0.0466 | 0.0455 | 0.0156 | 0.0082 | 0.0004 | 91.0 |
| 5 | 1 | 3:1 |  | 0.0538 |  |  | NMD |  |
| 6 | 1 | 6:1 | 0.0451 |  | 0.0148 |  |  | 57.6 |
| 6 | 1 | 6:1 | 0.0602 |  | 0.0217 |  |  | 52.8 |
| 7 | 1 | 6:1 | 0.1069 |  | 0.0205 |  | 0.0033 | 68.7 |
| Average |  |  | 0.0592 | 0.0438 | 0.0146 | 0.0088 | 0.0019 | 75.64 ^2^ |
| SEM |  |  | 0.0098 | 0.0023 | 0.0013 | 0.0017 | 0.0008 | 5.03 ^2^ |

^1^NMD = No measurable decay; Blank = No sample at that condition

^2^Average E_a_(decay) was determined by the best-fit slope of all data plotted on an Arrhenius plot as determined by a non-linear regression algorithm using Graphpad Prism software. SEM = Standard Error of Mean
